# Effects of semantic clustering and repetition on incidental vocabulary learning

**DOI:** 10.3389/fpsyg.2022.997951

**Published:** 2022-09-29

**Authors:** Mercedes Pérez-Serrano, Marta Nogueroles-López, Jon Andoni Duñabeitia

**Affiliations:** ^1^Departamento de Lengua Española y Teoría de la Literatura, Universidad Complutense de Madrid, Madrid, Spain; ^2^Departamento de Filología, Comunicación y Documentación, Universidad de Alcalá, Alcalá de Henares, Spain; ^3^Centro de Investigación Nebrija en Cognición (CINC), Universidad Nebrija, Madrid, Spain; ^4^Department of Languages and Culture, The Arctic University of Norway, Tromsø, Norway

**Keywords:** incidental learning, vocabulary learning, foreign language, semantic clustering, frequency of occurrence

## Abstract

The present study intended to investigate, first, the impact of semantic clustering on the recall and recognition of incidentally learned words in a new language, and second, how the interaction between semantic clustering and frequency of occurrence may modulate learning. To that end, Spanish university students watched an intentionally created video which contained Spanish target words that were either semantically related to others of the set, or not semantically linked at all. Furthermore, frequency of appearance changed among target words (1|4|8). All these words were paired with pseudowords that appeared as on-screen text during the videos. Participants were completely naive to the phases and the procedure of the experiment. After viewing the video, participants completed a recall test and a recognition test. Results showed that words presented in semantically unrelated categories were better recalled and better recognized than those presented in semantic clusters, especially when the words were presented more often.

## Introduction

Incidental vocabulary learning occurs when learners are not forewarned that a vocabulary test will follow (Hulstijn, 2001), and thus such learning takes place as the by-product of a meaning-focused task (Wesche and Paribakht, 1996; Ellis, 1999; Chen and Truscott, 2010); whereas intentional vocabulary learning takes place through exercises and activities (such as flash cards, fill in the blanks, or matching exercises) that are designed to explicitly focus students on learning given words (Webb, 2020b). It is generally acknowledged that incidental learning is an essential component of second language (L2) vocabulary development (Webb and Nation, 2017). For a word to be learnt, either incidental or intentionally, a single exposure to the target item is not always sufficient. In fact, the frequency of exposure to a word has been proved to promote vocabulary learning with audiovisual input (Peters et al., 2016; Malone, 2018; Peters and Webb, 2018), so that the more times users are exposed to a word, the more likely they are to recognize it and remember it.

Another variable that, together with frequency of occurrence, may have an impact on vocabulary learning is the way new vocabulary is presented. In the field of foreign language teaching, vocabulary has been traditionally taught in semantic groups consisting of words that are arranged together according to the semantic field and the syntactic word class they belong to (e.g., Marzano and Marzano, 1988). Despite this common practice in second language pedagogy, previous studies do not find agreement on whether this type of teaching causes interference or not in the learning of the target words due to the semantic similarity of such linguistic units. The present study focuses on the effects of semantic clustering on vocabulary learning as well as on the interaction between semantic clustering and frequency of occurrence, as such aspects have not been thoroughly explored in incidental learning situations.

The issue of incidental vocabulary learning in a second language has received considerable critical attention. While most research has focused on reading (Horst et al., 1998; Pellicer-Sanchez and Schmitt, 2010; Sánchez-Gutiérrez et al., 2019) and some on listening (van Zeeland and Schmitt, 2013; Uchihara et al., 2021), recent evidence suggests that vocabulary can also be learnt incidentally through viewing short videoclips (Montero Perez et al., 2014) and single full length television programs (Peters and Webb, 2018). Videos are considered an appropriate source of vocabulary learning since they provide both ecologically valid learning situations, as well as repeated encounters with low-frequency words through a relatively small amount of viewing (Webb and Rodgers, 2009). Additionally, research has shown that vocabulary is better learned when learners are exposed to simultaneous channels of information, namely, the auditory discourse, the written captions, and the visual images (Bisson et al., 2013; Rodgers, 2018; Pérez-Serrano et al., 2021).

There is robust evidence that words must be encountered many times in the input-be it written, aural or bimodal-in order for considerable gains to happen. Thus, frequency of occurrence is a critical factor that modulates incidental vocabulary learning, also with audiovisual input (Peters et al., 2016; Peters and Webb, 2018). As shown by Peters et al. (2016), there is a positive correlation between vocabulary growth and frequency of occurrence. In this line, on-screen text aids have also been shown to provide benefits for learners in terms of vocabulary learning depending on their frequency of appearance (Malone, 2018). Apart from the frequency of occurrence of the target items in the input, existing research recognizes the crucial role played by other critical factors in vocabulary growth. Vocabulary size (Peters and Webb, 2018; Montero Perez, 2020) and working memory skills (Montero Perez, 2020) are some of the learner-related factors with an influence on learning, while word relevance, cognateness (Peters and Webb, 2018) and the orthotactic pattern of the words (Pérez-Serrano et al., 2021) are some of the word-related factors that have been identified so far. However, there is one unexplored factor that is expected to modulate incidental vocabulary development and that has been consistently manipulated in educational contexts: the semantic relation between the target words presented in the input.

In L2 instructed settings, it is common to find that vocabulary is presented semantically clustered. Following Tinkham (1997), a semantic cluster contains words pertaining to the same semantic field (e.g., arm, leg, foot, hand, shoulder, etc.) which fall under the superordinate concept (e.g., parts of the body). Words semantically clustered also present syntactic similarities, since they all belong to the same syntactic word class. Importantly, this practice has originated some scientific debate around whether presenting words in semantic clusters promotes or hinders vocabulary learning, since empirical evidence remains inconclusive (see Bürki et al., 2020, for review). Behind those who defend the detrimental effects of semantic clustering on vocabulary learning, one can find the Interference theory and Distinctiveness theory. Interference theory (Higa, 1963; Baddeley, 1990) hypothesizes that similarity between language items to be learned increases the difficulty of learning and remembering such items (see Crowder, 1976). In line with this and focusing on the differences rather than on the similarities, the Distinctive hypothesis essentially states that differentiated language items are easier to learn (Hunt and Elliot, 1980; Hunt and Mitchell, 1982). In other words, according to these theories, semantic clustering impedes vocabulary learning because it causes interference between similar meanings of related words and creates competing memory traces. According to this, lexical items should then be presented in a non-related way, allowing learners to differentiate the target items. Based on these two theories, many researchers argue against semantic clustering as they claim that accessing semantically related words at the same time makes learning more difficult (e.g., Baddeley, 1997; Waring, 1997; Nation, 2000, 2013; Folse, 2004; Schmitt, 2007, 2010; Nation and Webb, 2011; Wilcox and Medina, 2013; Barcroft, 2015; Korochkina et al., 2021).

In sharp contrast, other researchers support that semantic clustering facilitates vocabulary learning (e.g., Grandy, 1992; Haycraft, 1993; Stoller and Grabe, 1995; Wharton and Race, 1999; Seal, 2001; Hashemi and Gowdasiaei, 2005; Hoshino, 2010), based on arguments from the Semantic field theory (Lehrer, 1974). This theory sustains the view that words with related meanings are closer in our mental lexicon. Thus, in order to mimic how vocabulary is organized in our minds, it should be presented in semantic sets (Nation, 2000). This way, such authors claim that it would be the most natural and logical way to present vocabulary in instructed settings. Additionally, as Nakata and Suzuki (2018) point out, the presumed difficulty posed by competing similar items in related sets might be beneficial in the sense that difficult items attract more attention and engagement from learners (Finkbeiner and Nicol, 2003).

Among the studies which found negative effects of semantic clustering on vocabulary learning, Tinkham (1993) compared the number of trials needed to recall target pseudowords previously presented with words in a semantically related and unrelated fashion. He found that significantly more trials were needed in the case of related words. Similarly, Wilcox and Medina (2013) found that semantically clustered (and not phonologically similar) words were more difficult to learn. Papathanasiou (2009) found a difference modulated by participants’ age and level of proficiency: semantic clustering would hinder learning for beginner adults, but not for children with an intermediate level. More recently, Korochkina et al. (2021) examined whether intended learning of new words was facilitated by presenting them together with words from the same semantic category, as opposed to with words from different semantic categories. Their experiment consisted of two sessions conducted on two consecutive days in which participants learned novel word-forms in German for familiar concepts, and were tested only in the second session through picture naming, translation and picture-word interference tasks. Items were intentionally displayed in groups of four belonging all four to either a related or unrelated context. As this experiment was framed in an explicit or intended learning scenario, participants were encouraged to practice and repeat words during the learning phase. The authors concluded that teaching new words together with other words from the same semantic category resulted in poorer accuracy in the learning phase and slower lexical access in the recall tasks. In this same line, Nakata and Suzuki (2018) studied the effects of semantic clustering on L2 vocabulary learning as well as the interaction between spacing and the semantic clustering of isolated lexical items in intentional learning. They tested whether spacing between target words enhanced the learning of semantically related words by reducing interference. While they did not find any significant differences between semantically related and unrelated sets in translation accuracy on the posttests, semantically related sets caused more within-set errors than did unrelated sets. They also found that in the massed group, semantically related items resulted in a larger number of interference errors than unrelated items on both the immediate and delayed posttests. As a consequence, their results also support the widely held view that semantic clustering should be avoided, at least when presented in massed sets.

In spite of the existing evidence, and probably as a consequence of generalized practices found in textbooks, there is still uncertainty whether semantic clustering is beneficial or not in educational contexts. While there is a growing body of research on the effects of semantic clustering, such studies exclusively focus on intentional learning scenarios. However, it is widely accepted that incidental vocabulary learning is central to lexical development (Webb, 2020a). The present study aims to address this issue by manipulating the semantic relatedness of new target words presented while participants viewed a subtitled video. Furthermore, considering that preceding research has shown that incidental vocabulary learning is modulated by the frequency of appearance of the items (Rott, 1999; Waring and Takaki, 2003; Pigada and Schmitt, 2006), in the current study, number of exposures was also manipulated. Interestingly, the interaction between both factors, namely, semantic clustering and frequency of occurrence, in incidental learning situations has not yet been thoroughly investigated. Thus, we designed a study to explore whether semantically unrelated words that are incidentally learned require less frequent occurrences to be learned.

## Materials and methods

### Participants

One hundred and two (70 women; mean age = 19) participants, aged between 18 and 22, were involved in the study. All of them were university students majoring in Education at University of Alcala and in Applied Languages and Linguistics at Complutense University of Madrid, and thus living in Spain, with Spanish as their L1. Before the experimental session, all participants gave their informed consent in accordance with guidelines approved by the Ethics and Research Committees of the Nebrija University. The experiment was implemented during synchronous online sessions with the researchers being present for problem solving if required. Participants were asked in advance to use an individual PC (rather than a tablet or a smartphone) and a headset.

### Design and stimuli

For the experiment, we used a repeated-measures design with Type of Item (semantically clustered|non-semantically clustered) and Number of Exposures (1|4|8) as within-subject factors. Following Pérez-Serrano et al. (2021), we intentionally created one video for this purpose, ensuring full control over the factors that could influence the results, such as the order of appearance of the targets, their particular linguistic features and the number of exposures to them. The soundtrack of the video consisted of 52 sentences in Spanish containing a total of 12 target words, as well as an opening and a closing sentence with no targets embedded. Each one of the 52 sentences contained a single mention to one of the Spanish targets. Half of the targets were semantically related, a set consisting of six coordinates as they all fell under the category of *fruits* (i.e., *grape, apple, pear, orange, mango, strawberry*). The use of coordinates, rather than synonyms or antonyms, is justified because previous studies have found significant effects of semantic relatedness using this type of semantically related targets in their designs (Tinkham, 1993, 1997; Finkbeiner and Nicol, 2003; Nakata and Suzuki, 2018). The other half were not semantically related at all, each one of them corresponding to independent categories such as tools, means of transportation, parts of the body, cutlery, clothes, tools, vehicles and animals (i.e., *finger, fork, skirt, hammer, motorbike, rabbit*). All the targets were controlled for L1-related factors, such as frequency of use in Spanish (zip. Mean 3.79; range 3.27–4.30), part of speech (all of them are nouns) and other semantic properties such as concreteness (mean 5.86; range 5.11–6.36).^1^ In each category, semantically related and unrelated, there were two items that appeared eight times, two that appeared four times and two that appeared only once. Each Spanish target word was paired with an invented two or three syllable pseudoword, that is, strings that do not exist in participants’ language, but that contain letter combinations that are orthotactically and phonotactically plausible (see Table 1). The invented words were created using Wuggy (Keuleers and Brysbaert, 2010). All of them were pronounceable in the L1 and contained legal combinations of letters in Spanish because previous literature has demonstrated that orthotactic markedness may affect the learning burden (Pérez-Serrano et al., 2021).

**Table 1:** Target words and pseudowords used in the experiment, together with the number of appearances of each of them (1, 4, 8), and their corresponding condition with regards to semantic clustering (related, unrelated).

Type of target	Target word	Pseudoword	Number of exposures
Semantically unrelated	martillo (hammer)	palvana	8
Semantically unrelated	moto (motorbike)	ina	8
Semantically unrelated	dedo (finger)	pima	4
Semantically unrelated	tenedor (fork)	nacarpa	4
Semantically unrelated	falda (skirt)	marno	1
Semantically unrelated	conejo (rabbit)	brena	1
Semantically related	uva (grape)	medo	8
Semantically related	manzana (apple)	parcallo	8
Semantically related	pera (pear)	veco	4
Semantically related	naranja (orange)	necador	4
Semantically related	mango (mango)	fesda	1
Semantically related	fresa (strawberry)	cocefa	1

The sentences contained objective information on the targets, such as “*The first motorcycle reached a speed of eighteen kilometers per hour*.” The texts were transformed into speech using an online software and the resulting audio clips were included in the videos using commercial video editing software. The verbal content was accompanied by supporting related visual information depicting the target words mentioned, which appeared on screen during the utterance of each sentence (see also Pérez-Serrano et al., 2021). The captions of the video appeared in the lower part of the screen and displayed the entire utterance, replacing the target words with the corresponding pseudowords in capital letters (“*The first INA reached a speed of eighteen kilometers per hour*”). The verbal input, except from the pseudowords, was in Spanish. Two different versions of the video clip were created in order to ensure that the place of appearance of the targets did not affect the results and thus to avoid cumulative semantic interference being a confounding variable. To do so, the order of appearance of the sentences in the audio files was randomized. The length of the videos was 6 min and 9 s. Each participant was randomly assigned one version of the video.

### Procedure

The experimental session lasted about 20 min in total and all the participants used an individual PC with a headset to complete the task. Materials were presented using Gorilla (Anwyl-Irvine et al., 2019). After the consent form and a short demographic questionnaire, participants watched their assigned version of the captioned video. No heads-up was given on the purpose of the experiment, the format of the videos or the tests that would come after. They were only instructed to pay attention to the screen, which ensured a true incidental learning situation where the association between the aural input (e.g., *motorbike*) and the on-screen text (e.g., the pseudoword *INA*) could easily occur. Once participants had watched the video, they completed a simple timed N-back filler task that was created to divert their attention from the content of the videos. At the end of it, participants took two tests aimed at assessing their gained productive and receptive knowledge of the correspondence between the new words (namely, the pseudowords) accidentally presented in the captions and the Spanish words played in the soundtrack (namely, the names of the fruits and the other entities). First, a recall task was presented. Students were given all the images previously displayed in the video one by one, and they were asked to type the pseudoword they thought that corresponded to each image. This task was followed by a recognition test, in which participants were provided with a pseudoword and 12 different images of the entities presented in the videoclips. The task consisted of choosing among all the images the one corresponding to the given pseudoword. This was done once for each of the 12 critical pseudowords in a random order for each participant.

## Results

**Figure 1:**
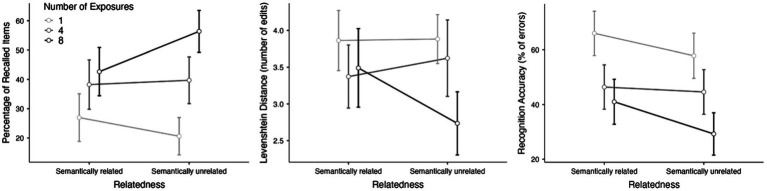
Marginal means plots of the recall accuracy (left), similarity of the recalled strings with the target items (middle) and recognition accuracy (right) split by condition. Error bars represent 95% confidence intervals.

A series of analysis of variance (ANOVAs) with Relatedness (semantically related|unrelated) and Number of Exposures (1|4|8) as within-subject factors were run to answer the research questions. The descriptive statistics of the different tasks split per condition are shown in Table 2 and Figure 1.

**Table 2:** Means and standard deviations (in parentheses) of the recall and recognition accuracy data (percentage of errors) per condition.

	Semantically related			Semantically unrelated		
	1	4	8	1	4	8
Recall accuracy (percentage of recalled items)	27 (41.4)	38.2 (42.9)	42.6 (41.9)	20.6 (32.5)	39.7 (40.6)	56.4 (36.3)
Levenshtein distance (number of edits)	3.86 (2.08)	3.37 (2.18)	3.49 (2.72)	3.88 (1.69)	3.62 (2.65)	2.74 (2.19)
Recognition accuracy (percentage of errors)	66.0 (41.2)	46.4 (41.3)	41.0 (41.9)	57.8 (42.1)	44.6 (41.6)	29.2 (39.4)

### Recall test

We first considered participants’ success or failure in typing the exact string of letters of the pseudoword. The ANOVA showed a main effect of Number of Exposures (*F*(2,202) = 56.29, *p* < 0.001, *η*^2^_partial_ = 0.358), signaling that learning gains increased as a function of repetitions, and an interaction between Number of Exposures and Relatedness (*F*(2,202) = 11.92, *p* < 0.001, *η*^2^_partial_ = 0.106). The main effect of Relatedness was not found to be significant (*F*(1,101) = 2.07, *p* = 0.15, *η*^2^_partial_ = 0.020). Holm-corrected pairwise comparisons indicated that the recall for items in the unrelated set increased with repetitions (1 vs. 4: *t*(101) = 5.72, *p* < 0.001; 4 vs. 8: *t*(101) = 4.58, *p* < 0.001), while this effect was much smaller for items in the related set (1 vs. 4: *t*(101) = 4.19, *p* < 0.001; 4 vs. 8: *t*(101) = 1.49, *p* = 0.419; see Figure 1). When exploring the differences between the two Relatedness levels for each of the levels of Number of Exposures, results showed that the only significant differences were found when items were repeated eight times (*t*(101) = 3.85, *p* < 0.001), with the other pairwise comparisons resulting not significant (*p*s > 0.10; see Figure 1).^2^

Since precise recall cannot be expected in an incidental learning task like the present one, we carried out further analysis on these data by computing the Levenshtein Distance (LD). LD is a string metric that measures the difference between two sequences, calculated by counting the number of edits (insertions, deletions or substitutions) required to change one string into the other. In this case, LD reveals a more accurate measure of recall, allowing us an estimation of the degree of similarity between the typed sequence and the expected one, with lower values meaning better performance. An ANOVA with the same factors and levels was carried out, and results revealed main effects of Number of Exposures (*F*(2,202) = 20.12, *p* < 0.001, *η*^2^_partial_ = 0.166) and Relatedness (*F* (1,101) = 4.28, *p* = 0.041, *η*^2^_partial_ = 0.041), and an interaction between the two factors (*F*(2,202) = 13.32, *p* < 0.001, *η*^2^_partial_ = 0.116). Post-hoc tests indicated that the number of edits needed to convert the typed string into the target decreased with repetitions, but this decrease was more marked for items in the unrelated set (unrelated set: 1 vs. 4: *t*(101) = 1.38, *p* = 0.682; 4 vs. 8: *t*(101) = 5.91, *p* < 0.001; related set: 1 vs. 4: *t*(101) = 3.93, *p* = 0.002; 4 vs. 8: *t*(101) = 0.84, *p* = 0.845). Similarly, the difference between Relatedness levels was only significant when items were repeated eight times (*t*(101) = 4.81, *p* < 0.001), and no significant pairwise contrasts were found when items were presented once or four time (*p*s > 0.52).

### Recognition test

Recognition data were analyzed following the same design. Participants showed an overall good level of accuracy in their recognition performance, since the mean percentage of errors was 47.5% in a task in which they had to choose among 12 possible candidates (see Figure 1 and Table 2). The ANOVA showed main effects of Number of Exposures (*F*(2,202) = 38.74, *p* < 0.001, *η*^2^_partial_ = 0.277) and Relatedness (*F*(1,101) = 10.84, *p* = 0.001, *η*^2^_partial_ = 0.097), but no interaction between the two factors (*F*(2,202) = 2.03, *p* = 0.134, *η*^2^_partial_ = 0.020). Elements presented in unrelated categories were better recognized than those presented in related categories, and recognition improved as a function of repetitions.

## Discussion

The current study provides the first known piece of evidence on the role of semantic clustering in incidental vocabulary learning. Previous studies evaluating the effects of semantic clustering on deliberate or intentional learning scenarios have reported inconsistent results on whether this way of presenting lexical items is beneficial for learners or not. Considering that the precise role of semantic clustering has not been explored in incidental learning scenarios, and that the manner in which this effect could interact with a repeatedly tested factor like the number of encounters with the target items has not been investigated to date, the present study was set to fill this gap.

The main question at test in this study sought to determine the effects of semantic clustering on incidental learning of words. In line with other studies like those by Finkbeiner and Nicol (2003), Papathanasiou (2009), and Tinkham (1993), our data consistently indicates that words presented in semantically related sets are more difficult to learn. Results from the recall test, considering both the success or failure in typing the exact learnt string as well as an index of similarity between the recalled string and the target one (e.g., the Levensthein distance), showed that learning is more likely to occur when words are presented with no semantic competitors, that is, in unrelated sets, especially when maximizing the exposure to the items to be learnt. Likewise, the analysis performed on the recognition data showed that learners are more successful in associating the correct name of the elements with their pictorial representation when the items had been presented in semantically unrelated sets during the learning phase. Such results are in line with Nakata and Suzuki’s study (2018), as they found that semantically related words resulted in a larger number of within-set errors than unrelated items, and thus, together these data support the view that semantic clustering should be avoided during learning. Our results are also consistent with the findings by Korochkina et al. (2021) who found that, as compared to the unrelated words, the categorically related items resulted in poorer naming accuracy in the learning phase, slower response latencies at the immediate recall tasks and greater semantic interference in the picture-word interference task. However, unlike Korochkina et al., in the current study the novel words were not presented blocked by group and learning context, providing additional evidence on learning scenarios that are closer to what a learner could experience in a real context. While in their case items belonging to the related group were presented in one block and those belonging to the unrelated context in another, in the present study, items were intermixed in the exposure and test phases.

Consistent with the literature on the role of the frequency of occurrence in incidental vocabulary learning, these data also demonstrated an effect of the number of exposures on the learning of the new words, showing that the more times a word is encountered, the more likely it is to be both recognized and recalled. Indeed, our results support preceding studies which confirm that frequency is closely tied to incidental vocabulary learning not only after exposure to written (Webb, 2007; Pellicer-Sanchez and Schmitt, 2010) and aural input (Brown et al., 2008; van Zeeland and Schmitt, 2013), but also with audiovisual input, both with captioned or uncaptioned videos (Montero Perez et al., 2013; Peters et al., 2016; Bairstow and Lavaur, 2017). With respect to the relationship between semantic relatedness among the items and the number of exposures to them during incidental learning, our recall data consistently showed that unrelated items benefited more from repeated encounters. Results indicated that recall of the unrelated words increased in a seemingly linear manner with repetitions (see at this regard the linear effect found in the mixed model analysis reported in Footnote 2), while this linear effect was less clear for semantically related words. As a result, we tentatively conclude that the beneficial effects of frequency in L2 vocabulary learning are more plausible to be observed when words are presented in semantically unrelated sets.

In this study, word learning is operationalized by remembering a completely unfamiliar and new label for a previously presented concept (in the images) and L1 word (in the soundtrack). Although unrelated words are not exempt from interference, in the related condition, the existence of semantic competitors is reinforced by the repeated appearance of the elements in the set. In this regard, Finkbeiner and Nicol (2003) suggested that this repetitive and residual activation of concepts and lemmas within the same semantic category could result in a much weaker connection between the new L2 lexical entry and the corresponding concept. With this in mind, we align with the proposal by Wilcox and Medina (2013) suggesting that the lack of effectiveness of semantic clustering could result from an inherent difficulty of the cognitive system to be fed with new words that have already been semantically preorganized, even though the ulterior way of storing accumulated vocabulary could be done in semantic fields. Thus, when designing courses, sessions or textbooks, it is worth considering the potentially detrimental effect of organizing the material in semantic neighborhoods.

An interesting aspect that should be noted is the fact that, unlike Nakata and Suzuki (2018), delayed posttest activities were not included in the current study. In the present study, the negative results found for the semantic clustered set of words in the relatively immediate recall and recognition test phases might be partially explained by the desirable difficulty framework (Bjork, 1999). This theory establishes that the interference caused by semantic clustered conditions makes learning more difficult, but that this difficulty may actually lead to better long-term retention. Further research should be aimed at investigating the effects of semantic clustering and number of encounters in incidental learning situations in the long term to test if undesirable effects of semantic relatedness fade with time.

Previous literature has also suggested that completely unfamiliar or poorly established words are more prone to the interference caused by semantic clustered presentation (Nation, 2000). If this is the case, a possible explanation for results in our study and those from Tinkham (1993, 1997) and Waring (1997) is the use of pseudowords. Using non-existing words ensures the lack of previous knowledge and mimics L2 novice learning situations. However, questions remain about how incidental or semantically related and unrelated words would be in more advanced learners, who are more likely to be familiar with at least one of the related words in the presented set.

There is abundant room for further progress in determining the role of individual differences in vocabulary learning. One relevant issue that emerges directly from our findings is whether a greater reliance on working memory in the learning phase (or enhanced working memory capacity) could moderate the negative effects of presenting new words in semantically related sets. The critical role of this cognitive mechanism has already been shown in incidental vocabulary learning through video (Montero Pérez 2020), and it has also been demonstrated that working memory has a greater impact when learning occurs intentionally (Bisson et al., 2021). Future research directly comparing intentional and incidental learning scenarios should be oriented at shedding light on the role of working memory in mitigating semantic relatedness effects. Lastly, in future investigations, it might be possible to rotate targets across the two conditions under study (namely, frequency of occurrence and semantic relatedness). Even though we created two versions of the videos in which the order of appearance of the targets was randomized and participants were also randomly assigned to them, we did control for the effect of extraneous variables since we did not rotate targets across conditions.

In sum, the present study measured the effects of semantic clustering and frequency of appearance on 12 new words presented incidentally and multimodally following a within-participants repeated measures design. Our results showed that words presented in categories which do not fall under the same semantic cluster are better recalled and better recognized than those corresponding to the same semantic neighborhood. Considering the pedagogical implications of these results, we propose that second language teachers and material designers should at least question presenting new vocabulary arranged in groups or lists of words that are semantically related, as the closeness in meaning between the items of each group may burden the learning of the items.

## Data availability statement

The raw data supporting the conclusions of this article will be made available by the authors, without undue reservation.

## Ethics statement

The studies involving human participants were reviewed and approved by Comité de Ética en Investigación (Universidad Nebrija). The patients/participants provided their written informed consent to participate in this study.

## Author contributions

MP-S, MN-L, and JD: conceptualization and writing—review and editing. MP-S: material creation and validation. JD: software design and development, data analysis, and supervision. MP-S and MN-L: data collection and writing—original draft preparation. All authors contributed to the article and approved the submitted version.

## Funding

This research has been partially funded by grants PID2021-126884NB-I00 from the Spanish Government, ISERI from the BBVA Foundation, and H2019/HUM-5705 from the Comunidad de Madrid.

## Conflict of interest

The authors declare that the research was conducted in the absence of any commercial or financial relationships that could be construed as a potential conflict of interest.

## Publisher’s note

All claims expressed in this article are solely those of the authors and do not necessarily represent those of their affiliated organizations, or those of the publisher, the editors and the reviewers. Any product that may be evaluated in this article, or claim that may be made by its manufacturer, is not guaranteed or endorsed by the publisher.

## Footnotes

^1^The lexical properties of the words were taken from Espal (Duchon et al., 2013).

^2^The same set of analysis was repeated using linear mixed effect modeling, including Relatedness and Number of Exposures (as a scaled and centered numerical variable) and their interaction as fixed effects and Participants as a random factor. Fixed effect omnibus tests are presented next, with the Satterthwaite correction method for degrees of freedom. When the main accuracy in the recall task was used as a dependent variable, the results replicated those obtained in the ANOVA, with a significant Number of Exposures effect (*F*(1,1,119) = 107.24, *p* < 0.001) and a significant Number of Exposures * Relatedness interaction (*F*(1,1,119) = 16.99, *p* < 0.001). The simple effects also showed that the effect of the Number of Exposures was larger for the unrelated (*F*(1,1,119) = 104.8, *p* < 0.001) than for the related set (*F*(1,1,119) = 19.4, *p* < 0.001). Parallel results were obtained when considering the Levenshtein Distance as the dependent variable from the recall test, with a significant Number of Exposures effect (*F*(1,1,119) = 36.79, *p* < 0.001) and a significant Number of Exposures * Relatedness interaction (*F*(1,1,119) = 11.05, *p* < 0.001). The simple effects also showed that the effect of the Number of Exposures was larger for the unrelated (*F*(1,1,119) = 44.08, *p* < 0.001) than for the related set (*F*(1,1,119) = 3.76, *p* = 0.053). The analysis of the data from the recognition test using linear mixed modeling showed a significant effect of Number of Exposures (*F*(1,1,134) = 96.80, *p* < 0.001) and of Relatedness (*F*(1,1,134) = 10.70, *p* = 0.001), with no interaction between the factors (*F* < 1 and *p* > 0.45), thus replicating the same results obtained in the ANOVA.
